# Railway track surface faults dataset

**DOI:** 10.1016/j.dib.2024.110050

**Published:** 2024-01-09

**Authors:** Asfar Arain, Sanaullah Mehran, Muhammad Zakir Shaikh, Dileep Kumar, Bhawani Shankar Chowdhry, Tanweer Hussain

**Affiliations:** a NCRA MUET, NCRA Condition Monitoring Systems Lab, Mehran University of Engineering and Technology, Jamshoro, Sindh, Pakistan; b Departmento de Ingeniería de Mecánica y Eficiencia Energética, Universidad de Malaga, 29016 Malaga, Spain

**Keywords:** Railway, Rail surface faults, Fault identification, Condition monitoring, Computer Vision

## Abstract

Railway infrastructure maintenance is critical for ensuring safe and efficient transportation networks. Railway track surface defects such as cracks, flakings, joints, spallings, shellings, squats, grooves pose substantial challenges to the integrity and longevity of the tracks. To address these challenges and facilitate further research, a novel dataset of railway track surface faults has been presented in this paper. It is collected using the EKENH9R cameras mounted on a railway inspection vehicle. This dataset represents a valuable resource for the railway maintenance and computer vision related scientific communities. This dataset includes a diverse range of real-world track surface faults under various environmental conditions and lighting scenarios. This makes it an important asset for the development and evaluation of Machine Learning (ML), Deep Learning (DL), and image processing algorithms. This paper also provides detailed annotations and metadata for each image class, enabling precise fault classification and severity assessment of the defects. Furthermore, this paper discusses the data collection process, highlights the significance of railway track maintenance, emphasizes the potential applications of this dataset in fault identification and predictive maintenance, and development of automated inspection systems. We encourage the research community to utilize this dataset for advancing the state-of-the-art research related to railway track surface condition monitoring.

Specifications Table

**Table:** 

Subject	*Mechanical Engineering and Railway Engineering*
Specific subject area	*Railway track surface faults detection/Visual inspection systems for railway tracks*
Data format	Raw images
Type of data	Tables, Images, Figures
Data collection	The data collection process is carried out using two EKENH9R cameras, each mounted on the both sides of the inspection vehicle. These cameras boast a respectable video recording speed of 120 frames per second (FPS) and offers a field of view (FOV) spanning approximately 14 inches. Their deployment allows for simultaneous video capturing from the both sides of the railway track while ensuring comprehensive coverage of the track's surface during the data collection. This dual-camera setup is essential for capturing various track conditions and anomalies under diverse lighting and environmental conditions. The 120 FPS rate is advantageous for capturing fine details in real-time. This makes the dataset, a valuable resource for the development and evaluation of advanced computer vision and Machine Learning models for railway track fault detection and identification. The collected video data is comprehensively pre-processed to ensure that only relevant frames are retained for further analysis. In this second step, use of Eq. (1) allows preserving all the pertinent information in the retained frames. Subsequently, the selected frames are manually labelled to indicate the presence of defect and particular type of defect.
Data source location	Pakistan Railway, Kotri Junction. Institution: NCRA Condition Monitoring Systems Lab, Mehran University of Engineering and Technology City: Jamshoro Country: Pakistan
Data accessibility	Repository name: Railway Track Surface Faults Dataset Data identification number: 10.17632/8hxtgyyxrw.2 Direct URL to data: https://data.mendeley.com/datasets/8hxtgyyxrw/2">https://data.mendeley.com/datasets/8hxtgyyxrw/2

## 1 Value of the Data

• Real-World Relevance: The collected dataset comprises a diverse range of conditions representing real-world railway track surface faults. Many of these faults have been caused by railway accidents. In that sense, this dataset provides a significant opportunity for addressing safety concerns and avoiding accidents in railway domain.
• Prototyping and Innovation: Researchers and engineers working in the railway domain will find this dataset as an invaluable resource for prototyping and developing novel solutions for railway track surface fault detection and classification. The rich and diverse dataset provides a real-world testing ground for innovative ideas and technologies.
• Benchmarking Cutting-Edge Methods: The established dataset provides a benchmark for the assessment of newly developed techniques, particularly in the domain of ML and DL. It serves as a reliable foundation for evaluating the performance and accuracy of ML/DL methods in railway track fault diagnosis. By using this dataset, researchers can validate the efficacy of their innovative methods, which ultimately allows for advancement in the state-of-the-art of railway infrastructure maintenance and safety.

## 2 Background

Railway track surface faults manifest on the railhead of railway tracks due to a multitude of operational and environmental factors. These faults can give rise to railway accidents by underestimating the critical importance of condition monitoring and predictive maintenance of railway infrastructure. To mitigate potential risks to railway operations and enhance safety, it becomes imperative to identify the specific types of railway track faults accurately [1,2]. Considering importance of the railway track faults detection and identification, this dataset is established which could serve for development of efficient condition monitoring systems for railway tracks.

## 3 Data Description

This paper reports an image dataset of railway track surface faults consisting of seven fault conditions including Grooves, Joints, Cracks, Flakings, Shellings, Spallings, and Squats. The details of the established dataset are given in Table 1.

**Table 1:** Details of dataset.

Dataset File	Number of Image	Description
Cracks	40	Extracted images of railway track surface cracks
Flakings	2830	Extracted images of railway track surface flakings
Joints	09	Extracted images of railway track joints
Shellings	230	Extracted images of railway track surface shellings
Spallings	291	Extracted images of railway track surface spallings
Squats	1842	Extracted images of railway track surface squats
Grooves	08	Extracted images of railway track surface grooves

The required number of frames is decided by implementing Eq. (1), which is twice of the ratio between assumed maximum speed of vehicle and FOV:

(1) $$frames\;required=\;2\times \frac{assumed\;maximum\;speed\;of\;vehicle\;\left(meter/sec\right)}{field\;of\;view\;\left(meter\right)}$$

## 4 Experimental Design and Data Acquition

The data is meticulously collected with the aim of advancement in the development of low-cost visual inspection systems, especially geared towards applications in developing countries, which cannot afford expensive advance systems for condition monitoring of railway infrastructure. Recognizing the cost constraints prevalent in these regions, the EKENH9R camera was strategically chosen as an affordable alternative to expensive high-speed cameras. This choice not only offers a low-budget- solution but also demonstrates the feasibility of achieving efficient and effective railway track surface fault inspection even with limited resources.

The established dataset represents a valuable and scholarly resource, suitable for the rigorous testing and evaluation of innovative methodologies aimed at enhancing the accuracy and efficacy of railway track fault identification. This dataset could be used for benchmarking purpose, as explored and validated in various research endeavors using different datasets, which serve as a foundation for the development and validation of cutting-edge techniques and technologies in the domain of railway infrastructure maintenance and safety [3], [4], [5].

**Fig. 1:**
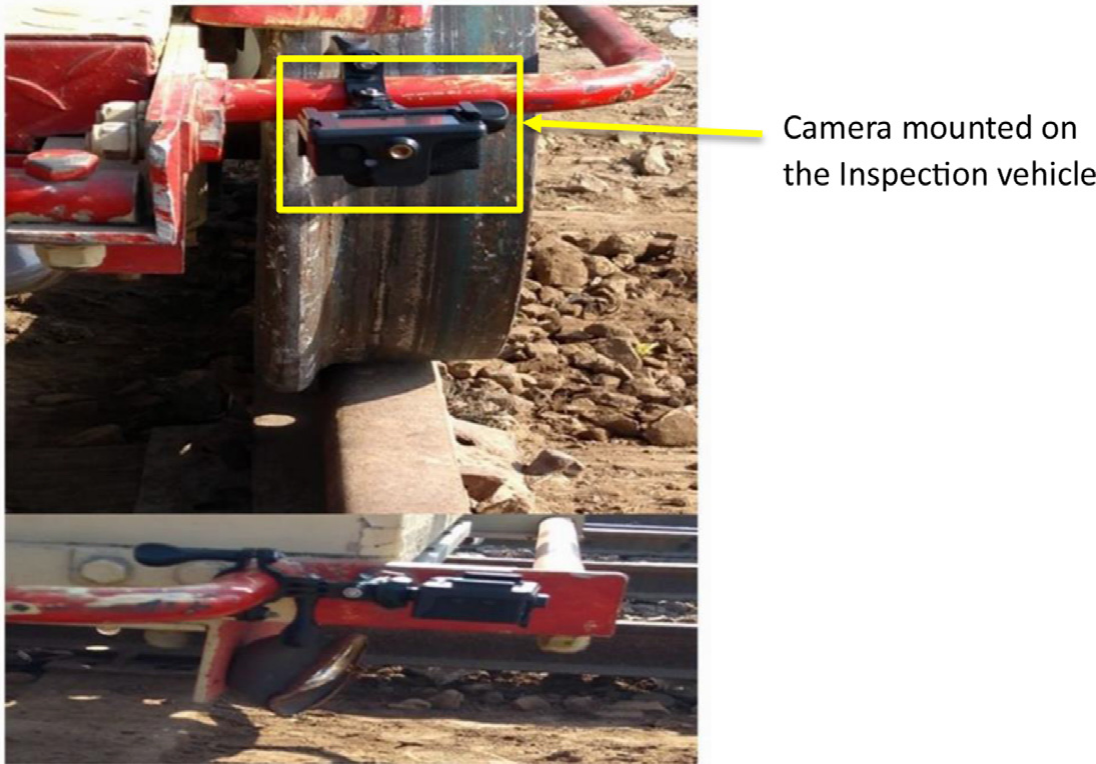
Experimental setup.

The dataset has been carefully collected through a structured approach, leveraging the deployment of two cameras installed on a specialized railway inspection vehicle as shown in Fig. 1. The dataset's establishment process involved the recording video of the railway track on the both sides during the inspection vehicle's operation. Subsequently, the video data has been pre-processed for removing irrelevant sections to ensure data integrity and precision. In the final phase of dataset preparation, frames have been carefully extracted from the processed video data. Each of these frames has been manually labelled to indicate the specific type of railway track fault present in the final image data.

During the data collection process, the EKENH9R cameras (specifications are given in Table 2) are consistently directed at the railhead throughout the inspection journey. The continuous and direct focus on the railhead allows toe capture a rich and detailed dataset, which ensures the reliability of the subsequent analyses and developments.

**Table 2:** Camera specifications.

Camera Model	Frames Per Second	Field of View
EKEN—H9R	120	14 inches

Furthermore, the data collection took place on the most heavily trafficked section of Kotri Junction, where surface faults are predominantly prevalent. This strategic selection of the data collection location adds significant value to the dataset. It represents a real world, high-traffic scenario, mirroring the operational conditions of many railway networks in developing countries. The collected dataset is thus highly relevant for addressing the challenges of railway infrastructure maintenance and safety in such conditions.

**Fig. 2:**
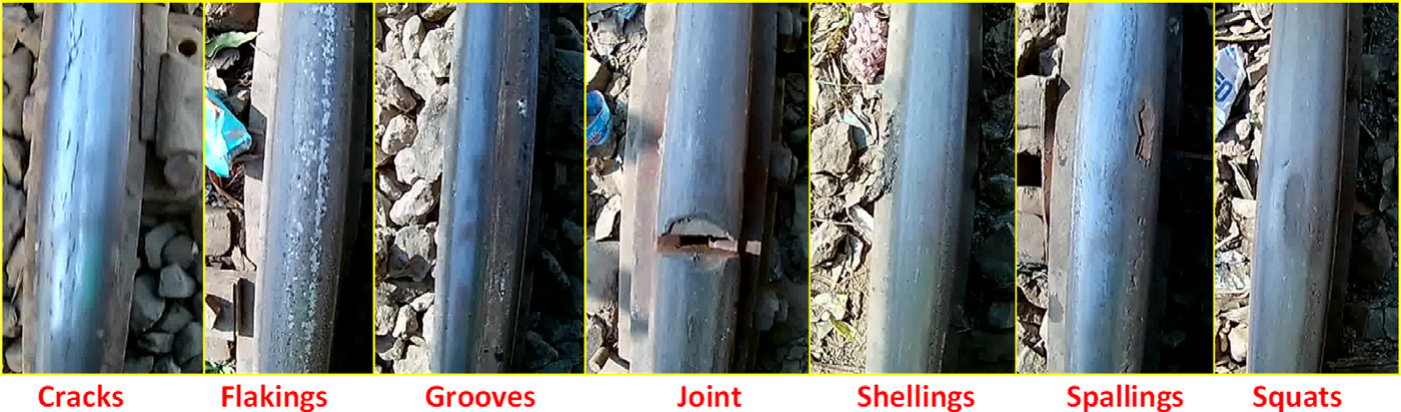
Types of the recorded railway track faults.

The inspection vehicle's maximum speed was limited to 20 km/h, ensuring that the data collected is favorable for precise and detailed inspection of railway track. This careful control of vehicle speed guarantees that the dataset provides clear and accurate insights into the nature of different surface faults on the railway track. The final dataset includes seven classes: Grooves, Joints, Cracks, Flakings, Shellings, Spallings, and Squats as shown in Fig. 2.

## Limitations

The established rail surface faults dataset, a valuable work for its insights into railhead conditions has certain limitations those should be considered for research employing this dataset. Firstly, the data collection process involved the use of an action camera capturing frames at a rate of 30 FPS. This high-speed capture introduces motion blur in certain instances, which potentially affects the clarity of fault representations.

Additionally, the absence of a controlled lighting setup poses a challenge, as some images may exhibit blurriness or glare, accompanied by reflections that may hinder fault detection accuracy. The primary focus of the dataset is on faults located directly on the railhead, neglecting potential anomalies along the sides of rails. This limitation may restrict the model's ability to generalize across the entire rail surface, which affects the comprehensiveness of a fault detection system.

Furthermore, the dataset solely includes fault classes without considering severity levels of different faults. The omission of severity assessment could limit the dataset's applicability in scenarios where the magnitude of fault impact is a critical factor. Thus, the scientific community should consider these constraints when using this dataset for their work.

## Ethics Statement

The current work meets the ethical requirements for publication in Data in Brief and does not involve human subjects, animal experiments, or any data collected from social media platforms. The NCRA MUET and NCRA Condition Monitoring Systems Lab, Mehran University of Engineering and Technology Jamshoro, Pakistan has given the consent that the datasets may be publicly released as part of this publication.

## Data Availability

Railway Track Surface Faults Dataset (Original data) (Mendeley Data)
